# Effect of miR-101 on the Proliferation and Apoptosis of Goat Hair Follicle Stem Cells

**DOI:** 10.3390/genes13061035

**Published:** 2022-06-09

**Authors:** Jingwen Qu, Xi Wu, Qiang Wang, Jian Wang, Xiaomei Sun, Dejun Ji, Yongjun Li

**Affiliations:** Key Laboratory for Animal Genetics & Molecular Breeding of Jiangsu Province, College of Animal Science and Technology, Yangzhou University, Yangzhou 225009, China; jwq9996@163.com (J.Q.); a17712698357@163.com (X.W.); junyouwang26@gmail.com (Q.W.); jianwang1223@163.com (J.W.); xiaomeikele@163.com (X.S.); djji@yzu.edu.cn (D.J.)

**Keywords:** Yangtze River Delta white goat, superior-quality brush hair trait, hair follicle stem cells, *DUSP1*, miR-101, proliferation, apoptosis

## Abstract

The Yangtze River Delta white goat is a rare goat species capable of producing high-quality brush hair. Dual specificity protein phosphatase 1 (*DUSP1*) may play a role in the formation of high-quality brush hair, as evidenced by our previous research. We investigated the potential mechanisms that regulate the proliferation and apoptosis of goat hair follicle stem cells. We particularly focused on the relationship between *DUSP1* and miR-101, which directly targets *DUSP1,* predicted and screened through bioinformatics websites. Then, fluorescence assays, flow cytometry, RT-qPCR, and Western blotting were used to investigate the effects of miR-101 on the proliferation and apoptosis of hair follicle stem cells. We found that miR-101 overexpression significantly decreased (*p* < 0.01) apoptosis and promoted the proliferation of hair follicle stem cells. Furthermore, the overexpression of miR-101 increased (*p* < 0.05) the mRNA and protein expression levels of the proliferation-related gene (*PCNA*) and anti-apoptotic gene (*Bcl-2*), and it decreased (*p* < 0.05) the mRNA and protein expression levels of the apoptotic gene (*Bax*). In conclusion, miR-101 can promote the proliferation of and inhibit the apoptosis of hair follicle stem cells by targeting *DUSP1*, which provides a theoretical basis for further elucidating the molecular mechanism that regulates the production of high-quality brush hair of Yangtze River Delta white goats.

## 1. Introduction

The Yangtze River Delta white goat, indigenous to China, produces high-quality brush hair, which is a unique raw material that is used to make top-grade writing brushes. Its unique superior-quality brush hair that is obtained mainly from the neck and ridge of the goat is valuable for many excellent characteristics, including its white color, straight peak, fine luster and elasticity [1]. Yangtze River Delta white goat brush hair of high quality, with prices ranging from 2500–3000 USD per kilogram, is popular in Japan, Korea, Singapore and other Southeast Asian nations influenced by Chinese culture, and it is typically a traditional Chinese export commodity [2]. The production of high-quality brush hair can be traced to the characteristic development of the development of hair follicles. The growth and development of hair follicles affect not only the growth, but also the quality of hair. Hair follicle development is a cyclic process, involving three stages: anagen, catagen and telogen, and it involves dynamic changes in gene regulation [3]. Dual-specificity phosphatase 1 (*DUSP1*), a two-way specific Sue/tyrosinase, is involved in inactivating different MAPK family isoforms [4], and it plays an indispensable role in cell cycle arrest and cell proliferation in tumor and normal cells [5,6]. *DUSP1* has been recognized to play a role in tumorigenesis and tumor progression, as well as the proliferation, differentiation and apoptosis of normal cells [7]. Interestingly, in our previous study, we found that *DUSP1* is significantly up-regulated in high-quality brush hair than in non-high-quality brush hair follicle cells [8,9]. Therefore, we hypothesized that *DUSP1* may play an important role in the regulation of the growth and development of hair follicles, and consequently, of hair quality as well.

MicroRNAs are short (20–24 nt) single-stranded, non-coding RNAs that bind to the 3′-UTR of target mRNAs, translation repression or degradation of target mRNAs, resulting in the silencing of the target gene [10]. Studies have demonstrated that miRNAs participate in a variety of physiological processes, including proliferation, cell differentiation, apoptosis and even tissue and organogenesis development [11,12]. To date, several studies have shown that many miRNAs, such as miR-218-5p [13], miR-let7a [14] and miR-214 [15], are essential for hair follicle development and regeneration in skin tissue. MiR-101, encoded by two precursor transcripts (miR-101-1 and miR-101-2), is considered a tumor suppressor, as it targets mRNAs of critical oncogenes or anti-oncogenes, and its loss is associated with the occurrence and progression of various diseases [16]. It was manifested that miR-101 tends to inhibit cell proliferation by downregulating the expression of the enhancers of zeste homolog 2 (EZH2) in lung cancer [17], bladder transitional cell carcinoma [18] and embryonal rhabdomyosarcoma [19]. Furthermore, the study of Wang et al. revealed that the upregulation of miR-101-3p promotes apoptosis and inhibits the viability of oral cancer cells [20]. However, the function and mechanism of goat miR-101 in goat HFSCs in the Yangtze River Delta white goat have rarely been explored. Therefore, in this study, we explored the relationship between miR-101 and *DUSP1*, particularly focusing on their role in the production of superior-quality hair in Yangtze River Delta white goats.

## 2. Materials and Methods

### 2.1. Isolation and Culture of Cells

Hair follicle stem cells (HFSCs) from Yangtze River Delta white goats were extracted from the skin of the neck-spine of newborn lambs 120 days after birth at the Haimen State Goat Farm (Haimen City, Jiangsu Province, China), which was described and verified in our previous study [21]. Briefly, approximately 2 cm^2^ of skin was separated from newborn lambs and soaked in 75% alcohol for 30 s, followed by ringing in phosphate-buffered saline (PBS) three times. Next, the tissue blocks were cut into 2 × 2 mm pieces and were digested with 0.25% trypsin at 37 °C for 90 min. Next, the hair follicles were isolated and digested for 30 min. Eventually, HFSCs were obtained through crushing and sieving, and they were subsequently verified by confirming CD49f and CD34 double [22]. The sorted HFSCs and HEK293T cells were individually cultivated in 6-well plates (Corning, New York, NY, USA) with Dulbecco’s modified Eagle’s medium (DMEM/F12), supplemented with 10% fetal bovine serum (FBS) and 2% penicillin–streptomycin at 37 °C in a 5% CO_2_ atmosphere.

### 2.2. Prediction of miRNA Targeting DUSP1

TargetScan (http://www.targetscan.org/vert_72, accessed on 1 October 2021), RNAhybrid (https://bibiserv.cebitec.uni-bielefeld.de/rnahybrid/, accessed on 1 October 2021) and miRDB software were employed to predict miRNAs and their binding regions that could target *DUSP1*, and eventually, miR-101 was selected for further research.

### 2.3. Synthesis of Primers

According to the NCBI nucleic acid database (https://www.ncbi.nlm.nih.gov/nuccore/, accessed on 1 October 2021), the CDS and 3’-UTR of goat *DUSP1* (registration number: XM_018065720.1) were obtained. All the primers were designed by Prime 5.0 software and the NCBI Primer-BLAST online website. *GAPDH* expression was used as the internal control. The primers for quantitative PCR were synthesized by Shanghai Bioengineering Co., Ltd. and are shown in Table 1. The Wt-*DUSP1*-miR101 and Mut-*DUSP1*-miR101 plasmids were constructed by the Gene Pharma of China.

### 2.4. Double Luciferase Reporter Assay

The wild-type 3′-untranslated region (UTR) of *DUSP1* mRNA with a putative miR-101 binding site was engineered into pmirGLO, a dual-luciferase miRNA target expression vector (Promega, Madison, WI, USA) doubly digested with SacI and XhoI to generate Wt-DUSP1-miR101 plasmid. The Mut-DUSP1-miR101 plasmid was constructed by displacing the binding sites of miR-101 from AGTACTGTA to CCGAAATTT. The recombinant plasmids identified by sequencing were called Wt-DUSP1 and Mut-DUSP1. Subsequently, 293T cells were plated in 24-well plates when the cells reached 60%~70% confluence, and they were co-transfected with miR-101 mimics or miR-NC and Wt-*DUSP1* or Mut-*DUSP1* plasmid for 24 h using Lipofectamine 2000 (Invitrogen) according to the manufacturer’s instructions. After transfection, the relative luciferase activity was determined using the Dual-Luciferase Reporter Assay System (Promega) according to the manufacturer’s instructions.

### 2.5. Construction and Transfection of Plasmid

The oligonucleotides of the negative control (NC), the miR-101 mimics, Inhibitor-NC and miR-101 inhibitors were synthesized by Gene pharma (Gene pharma Co., Ltd. Suzhou, China). For cell transfections, transient transfections were then performed using lipofectamine 3000 (Cat: L3000015, Invitrogen, CA, USA) according to the instructions of the manufacturer. In brief, after the stem cells were cultured for 24 h and reached 60%–70% confluence, miR-101 overexpression and knockdown in HFSCs were accomplished by transfecting with 100 nM miR-101 mimics and 200 nM miR-101 inhibitors, respectively. Total RNA or protein was harvested 48 h following transfection.

### 2.6. Quantitative Real-Time PCR

Total RNA was extracted from cultured HFSCs using the Trizol kit (Cat: 9109, Takara, Tokyo, Japan). The concentration and purity of extracted RNA were verified using Nanodrop spectrophotometry (Thermo Scientific, Shanghai, China), and the RNA was subsequently stored at −80 °C. Next, cDNA was synthesized using the EasyScript One-step gDNA Removal and cDNA Synthesis SuperMix kit (Cat: AE311, Transgen, Beijing, China) in accordance with the manufacturer’s instructions. The synthesized cDNA as a template was then used for the real-time PCR quantification of mRNAs on an ABI 7500 Real-Time PCR System (Applied Biosystems, Foster City, CA, USA) in light of the instructions of PerfectStartTM Green qPCR SuperMix Kit (Cat: AQ602, Transgen, Beijing, China) and the relative gene expression levels computed by the 2^−^^ΔΔ^^Ct^ method. The related primers in this study were synthesized by Sangon Biotech (Shanghai, China), which are shown in Table 1.

### 2.7. EdU Incorporation Assay

For evaluating the proliferation potential of goat HFSCs, the Cell-Light TM EdU cell proliferation kit (Cat: C103101-1, RiboBio, Guangzhou, China) was used. Briefly, HFSCs were seeded in a 24-well culture plate at a density of 1 × 10^5^ cells/well and were transfected with miR-101 oligo-nucleotides (Table 2) for 6 h. Then, the cells were transferred into a complete medium containing 10 μM 5-ethynyl-2′-deoxyuridine (EdU) reagent and were incubated for 24 h at 37 °C and 5% CO_2_. Next, the cells were fixed with 4% paraformaldehyde and were incubated with 1 × Apollo567 solution, and the cell nuclei were stained with 1 × Hoechst33342 solution for 30 min in the dark. Eventually, the fluorescence intensity of cells was observed immediately under a Leica fluorescence microscope (Leica, Wetzlar, Germany), and the percentage of EdU-positive nuclei (red) relative to the blue fluorescent nuclei was calculated using the Image J software.

### 2.8. Apoptosis Assay

After transfection, the apoptosis of different treatment groups was analyzed according to the instructions of the Annexin V-FITC apoptosis detection kit (Cat: C1062M, Beyotime, Shanghai, China). Flow cytometry (FACSAria SORP, BD Biosciences, CA, USA) was used to detect apoptosis rate. Flowjo software was used for the apoptosis rate.

### 2.9. Western Blotting

Total proteins were extracted 48 h after transient transfections according to the manufacturer’s instructions of efficient RIPA tissue/cell lysis buffer (Cat: R0010, Solarbio, Shanghai, China) containing 1% PMSF (Cat: P0100, Solarbio, Beijing, China) and were subjected to Western blotting. In brief, 10 μg proteins was separated by 10% or 12% SDS-polyacrylamide gel electrophoresis. Next, the membranes were rinsed three times with 1 × TBST (Tris-buffered saline buffer supplemented with Tween 20) for 10 min each and were blocked with 5% skim milk (Sangon Biotech, Shanghai, China) for 1 h at room temperature. Subsequently, the membranes were incubated at 4 °C overnight with appropriate dilutions of specific primary antibodies, namely, proliferating cell nuclear antigen (PCNA) (MW: 29kDa, Abcam, Cambridge, United Kingdom, 1:1000 dilution), cyclin dependent kinase 1 (CDK1), (MW: 34 kDa, Abcam, Cambridge, United Kingdom, 1:1000 dilution), cyclin D2 (CCND2) (MW: 33 kDa, Abcam, Cambridge, United Kingdom, 1:1000 dilution), BCL2 associated X, apoptosis regulator (Bax) (MW: 21 kDa, Abcam, Cambridge, United Kingdom, 1:5000 dilution), BCL2 apoptosis regulator (Bcl-2) (MW: 26 kDa, Proteintech, Rosemont, IL, United States, 1:1000 dilution) and β-actin (MW: 42 kDa, Bioworld, Nanjing, China, 1:5000 dilution). After washing, the membranes were incubated for 1 h at room temperature with the second antibodies, namely, goat-specific anti-rabbit IgG and goat-specific anti-mouse IgG ((Bioworld, Nanjing, China, 1:5000 dilution)). The immunoreactive bands were visualized using an ECL system (Biosharp, Hefei, China) and were analyzed using a FluorChem FC3 system (Protein-Simple, CA, USA).

### 2.10. Statistical Analysis

All data in this study were obtained from at least three independent experiments and are presented as the mean ± standard error (Mean ± SEM). The SPSS 24.0 (SPSS, Inc., Chicago, IL, USA) statistical software was used for one-way ANOVA analysis and independent-samples *t*-tests. The graphs were drawn using the GraphPad Prism 7.0 software. Differences are considered statistically significant when shown with an asterisk character. * means that *p* < 0.05 and that the difference is significant; ** means that *p* < 0.01 and that the difference is extremely significant.

## 3. Results

### 3.1. DUSP1 Is a Direct Target of miR-101

**Figure 1:**
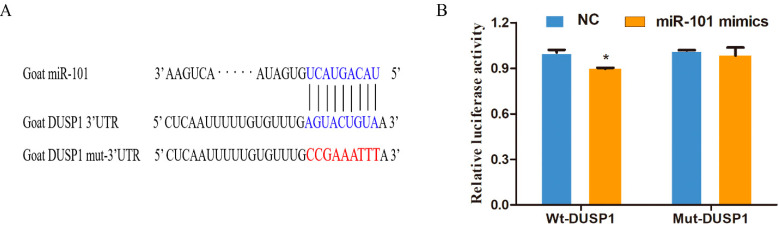
MiR-101 can directly target the 3′UTR of the *DUSP1* gene in hair follicle stem cells: (**A**) The predicted binding sites of miR-101 and *DUSP1*; the bases marked with blue represent the wild-type, and those in red represent the mutant-type; (**B**) Detection of dual-luciferase activity after co-transfection of NC or miR-101 with wild or mutant-type *DUSP1.* No asterisk, *p* > 0.05; *, *p* < 0.05.

To confirm the biological regulation of miR-101 and *DUSP1*, it was discovered that miR-101 targets *DUSP1* via a conserved region inside the 3′-UTR of *DUSP1* (Figure 1A) using algorithm prediction. Subsequently, we verified the relationship between miR-101 and *DUSP1* using a luciferase activity assay. As shown in Figure 1B and Figure S1, luciferase activity was significantly reduced in 293T cell lines transfected with miR-101 mimics compared to those transfected with miR-NC in Wt-*DUSP1* plasmid (*p* < 0.05), whereas the difference was not significant in Mut-DUSP1 plasmid(Figure S2). These results confirm that miR-101 targets *DUSP1* in hair follicle stem cells to regulate their function.

### 3.2. Effect of miR-101 on the Proliferation of HFSCs

**Figure 2:**
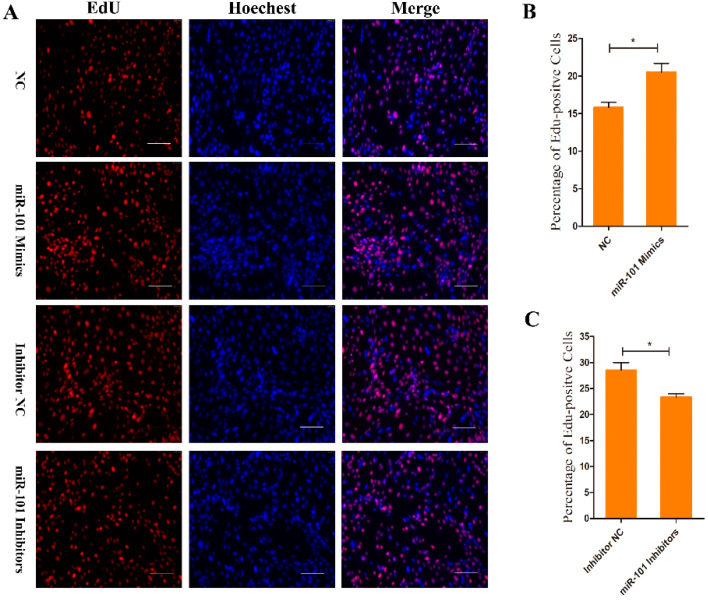
EdU assay of HFSCs proliferation after transfection with NC, goat miR-101 mimics, inhibitor NC and goat miR-101 inhibitors: (**A**) Representative images after EdU assay of HFSCs at 24 h after transfection with NC, goat miR-101 mimics, inhibitor NC and goat miR-101 inhibitors. Bars = 100 μm; (**B**,**C**) Quantification of EdU-positive cells. The proportion of EdU-positive cells was calculated as (EdU-positive cells/Hoechst-stained cells) × 100%. No asterisk, *p* > 0.05; *, *p* < 0.05.

The proliferation potential of Goat HFSCs was determined using an EdU assay. Compared to the NC counterparts, the overexpression of miR-101 (miR-101 mimic-treated group) significantly enhanced the ratio of EdU-positive stem cells (*p* < 0.05), whereas the proportion was remarkably decreased after inhibiting the expression of miR-101 (miR-101 inhibitor-treated group) compared with inhibitor NC counterpart (*p* < 0.05), as shown in Figure 2B,C. The results above indicate that the overexpression of miR-101 can significantly promote the proliferation of hair follicle stem cells.

### 3.3. Effect of miR-101 on the Apoptosis of HFSCs

**Figure 3:**
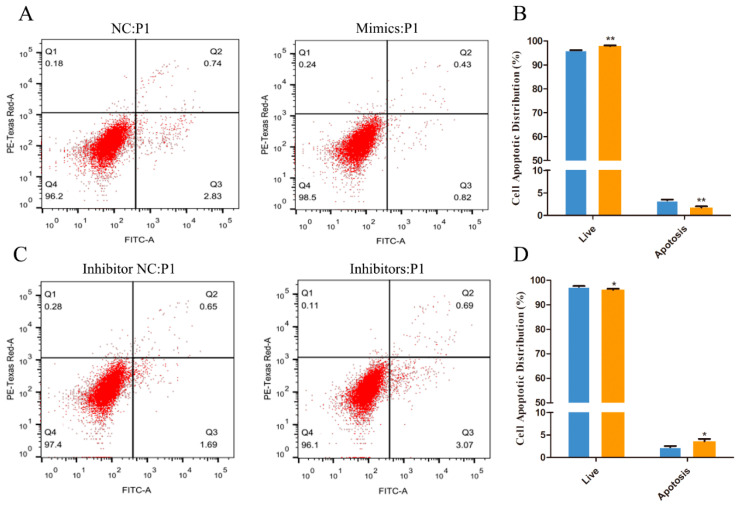
The Annexin V-FITC/PI assay was performed to quantify HFSC apoptotic distribution: (**A**,**B**) The apoptosis levels of the cells transfected with NC and miR-101 mimics were tested by Annexin V-FITC/PI kit and flow cytometry; (**C**,**D**) The apoptosis levels of the cells transfected with inhibitor NC and miR-101 inhibitors were tested by Annexin V-FITC/PI kit and flow cytometry. Note: NC: negative control; Mimics: goat miR-101 mimic; INC: inhibitor NC; IN, inhibitor: goat miR-101 inhibitors. No asterisk, *p* > 0.05; *, *p* < 0.05; **, *p* < 0.01.

The Annexin V-FITC and PI double staining assay was performed to quantify HFSC apoptosis. The proportion of the specific cell population at various treatment groups is represented in Figure 3. As the result show, compared to the NC group, upregulating the expression of miR-101 can significantly increase the rate of living cell (98.5% vs. NC: 96.2%) and decrease the total apoptosis percentage (1.25% vs. NC: 3.57%) (*p* < 0.01) (Figure 3A,B). However, the total apoptosis rate of the inhibitor group (4.76%) was significantly higher than that of the inhibitor NC group (2.34%) (*p* < 0.05), and the living cell rate of the inhibitors group (96.1%) significantly declined (*p* < 0.05) compared with the inhibitor NC group (97.4%) (Figure 3C,D). The results above indicate that overexpression of miR-101 can significantly inhibit the apoptosis of hair follicle stem cells.

### 3.4. Effect of miR-101 on the Expression Levels of Proliferation and Apoptosis-Related Genes and Proteins

**Figure 4:**
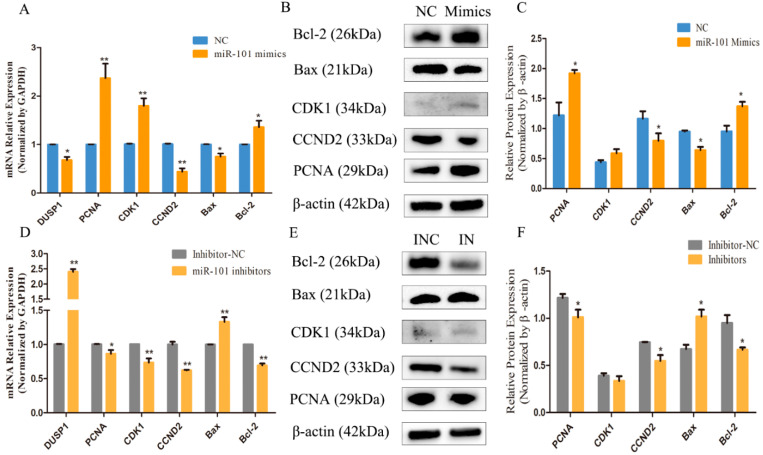
Effect of miR-101 on the expression levels of proliferation and apoptosis-related genes and proteins: (**A**,**D**) The mRNA expression of *DUSP1*, proliferation-related genes (*PCNA*, *CDK1* and *CCND2*) and apoptosis-related genes (*Bax* and *Bcl2*) at 48 h, after being transfected with NC, miR-101 mimics, inhibitor NC and miR-101 inhibitors, were determined by RT-qPCR. *GAPDH* was used as an internal control; (**B**,**E**) Protein expression of proliferation-related genes (*PCNA*, *CDK1* and *CCND2*) and apoptosis-related genes (*Bax* and *Bcl2*) at 48 h, after being transfected with NC, mimics, INC and inhibitors, were determined by Western blotting; (**C**,**F**) The relative expression of related proteins from each group are shown as the mean ± SEM, which were obtained from at least three independent replicates (independent-samples t-tests). No asterisk, *p* > 0.05; *, *p* < 0.05; **, *p* < 0.01.

To investigate the effect of miR-101 on the expression levels of genes related to the proliferation and apoptosis of HFSCs, the expression levels of the proliferation-related genes (*PCNA*, *CDK1* and *CCND2*) and apoptosis-related genes (*Bax* and *Bcl2*) were determined by RT-qPCR and Western blotting. As shown in Figure 4A, compared with the negative control group, the overexpression of miR-101 significantly decreased (*p* < 0.05) the mRNA expression of its target gene *DUSP1*, and the miR-101 mimic treatment significantly increased (*p* < 0.01) the mRNA expression levels of proliferation-related genes (*PCNA* and *CDK1*) and significantly decreased (*p* < 0.01) the mRNA expression of *CCND2*. Moreover, the overexpression of miR-101 led to a significant decline (*p* < 0.05) in the expression levels of pro-apoptotic genes (*Bax*) and an obvious augmentation (*p* < 0.05) of the expression levels of anti-apoptotic genes (*Bcl-2*). Combined with the Western blot test, the impact of miR-101 on proliferation and apoptosis-related protein expression was further determined. As shown in Figure 4B,C, compared with the control group, the relative protein expression of proliferation-related genes (*PCNA*) in the miR-101 mimic group was significantly enhanced (*p* < 0.05), and the relative protein expression of *CDK1* tended to rise despite the lack of a difference. Moreover, concerning another proliferation-related gene (*CCND2*), its expression level was lower in the enhanced miR-101 treatment than it was in the negative control. Furthermore, the relative expression of pro-apoptotic genes (*Bax*) was dramatically reduced (*p* < 0.05), but the relative expression of anti-apoptotic genes (*Bcl-2*) was significantly raised (*p* < 0.05).

The results shown in Figure 4D reveal that the downregulation of the expression of miR-101 can significantly increase (*p* < 0.01) the mRNA expression of its target gene *DUSP1* and can decrease (*p* < 0.05) the expression of the proliferation-related genes, *PCNA*, *CDK1* and *CCDN2*, and the anti-apoptotic gene (*Bcl-2*), and it can greatly increase (*p* < 0.01) the expression of pro-apoptotic genes (*Bax*) and the *Bax*/*Bcl-2* ratio compared with the negative control group. Furthermore, the results of the Western blot assay presented in Figure 4E,F manifest that the inhibition expression of miR-101 led to significantly decreasing the expression levels of proliferation-related genes (*PCNA* and *CCDN2*) and *Bcl-2*, and it significantly increased the expression levels of the Bax protein. In general, the results of RT-qPCR and Western blotting are consistent. Therefore, it can be speculated that the overexpression of miR-101 can enhance hair follicle stem cell growth and prevent hair follicle stem cell apoptosis.

## 4. Discussion

The results in our present study demonstrate that miR-101 promotes proliferation but inhibits the apoptosis of hair follicle stem cells. However, the mRNA and protein expression of *CCND2*, a proliferation-related gene in the cell cycle, tended to be decreased (*p* < 0.05). The cell cycle in most eukaryotic cells is a complex process, including a series of coordinated events, namely, preparation for cell growth (G1 phase), the replication of genetic material (S phase), the segregation of duplicated chromosomes (G2 phase) and cell division (M phase), which is regulated by cyclins, cyclin dependent kinases (CDKs) and CDK inhibitors [23]. The three D type cyclins (*CCND1*, *CCND2* and *CCND3*) that modulate the cell cycle by engaging and activating CDKs, such as *CDK4* and *CDK6* and CDK-cyclin D complexes, are essential for G1 entrance [24]. Cyclin A combines with *CDK1* in the late G2 and early M phase, and its protein levels stay constant throughout the cell cycle. Promoting entrance into mitosis is further governed by cyclin B in the complex with *CDK1* [25,26]. These reports can verify our findings that enhanced miR-101 led to the reduction in the expression level of *CCDN2* in the overexpression in the miR-101 treatment compared with the negative control group. It has been reported, furthermore, that miR-101 suppresses the expression of *CCND2* in *H. pylori* related to gastric cancer [27], which is in accordance with our results.

*DUSP1* as a phosphatase can dephosphorylate threonines and/or tyrosines of client proteins and inactivate mitogen-activated protein kinase kinase kinase 1 (*MAP3K1*), a member of the mitogen-activated protein kinase (*MAPK*) family and a signal transduction modulator to trigger MAPKs through dephosphorylation [28,29]. The activated *MAP3K1* (also called p42/p44 MAPK) cascade promotes *DUSP1* activity, which in turn attenuates *MAP3K1*-dependent events [30]. Thus, *DUSP1* influences MAPK signaling and its targets through a negative feedback control mechanism [31,32]. Studies have shown that *DUSP1* is specifically expressed, which may be associated with MAP3K1 phosphorylation [33], and reinforced *DUSP1* expression results in the suppression of proliferation and altered cell apoptosis rates by suppressing the MAPK/ERK cascade, p-ERK, p-Elk-1 and Egr-1 [34].

Our previous results have manifested that some major genes are involved in the production of superior goat brush hair, including *MAP3K1*, *CMTM3* (CKLF-like MARVEL transmembrane domain-containing family 3), *DUSP1* (dual specificity protein phosphatase 1) and *DUSP6* (dual specificity protein phosphatase 6) [35]. We also found that *MAP3K1* expression was much higher in the skin tissue of superior-quality brush hair than their counterparts in normal-quality brush hair, and silencing *MAP3K1* can greatly reduce proliferation and induce the death of superior goat hair follicle stem cells [36]. In this study, we confirmed that miR-101 can directly target *DUSP1* by bioinformatics predictions and dual-luciferase reporter gene assays. Furthermore, the overexpression of miR-101 can facilitate proliferation and inhibit the apoptosis of hair follicle stem cells. Therefore, we postulate that miR-101 targets *DUSP1* and reduces its expression, which, in turn, reduces the dephosphorylation of *MAP3K1*, and phosphorylated *MAP3K1* serves as a positive regulator of proliferation and cell survival and inhibits the apoptosis of hair follicle stem cells in vitro. Furthermore, accumulating studies have illustrated that miR-101, as a negative regulator, can target *DUSP1* and downregulate its expression [37,38,39,40].

Interestingly, previous high-throughput RNA-Seq results of our study have manifested that both *MAP3K1* and *DUSP1* are upregulated in skin samples of Yangtze River Delta white goats, which can generate high-quality hair compared with those that produce non-high-quality hair [8]. The potential molecular mechanisms behind this may likely include p-MAP3K1 upregulating the expression of *DUSP1*, which is vital for high-quality brush hair production. We will further explore the molecular mechanisms of *DUSP1*/*MAP3K1* and how they regulate the production of superior-quality hair in Yangtze River Delta White in vitro and in vivo.

Taken together, this study identifies miR-101 as a positive regulator in the proliferation of hair follicle stem cells related to the production of high-quality brush hair, and downregulating miR-101 results in the inhibition of the proliferation and promotion of apoptosis of hair follicle stem cells. The above findings demonstrate that miR-101 may play an important role in regulating the formation of superior-quality brush hair traits in Yangtze River Delta white goats.

## Figures and Tables

**Table 1:** Quantitative real-time PCR primer pairs.

Genes	Gene ID	Primer Sequence (5′–3′)
*GAPDH*	100860872	F: AGGTCGGAGTGAACGGATTC
		R: CCAGCATCACCCCACTTGAT
*DUSP1*	539175	F: CCACCACCACCGTCTTCAACTTC
		R: GCTGGGAGAGGTCGTGATAGGG
*PCNA*	102172276	F: ATCAGCTCAAGTGGCGTGAA
		R: TGCCAAGGTGTCCGCATTAT
*CDK1*	10086361	F: AGATTTTGGCCTTGCCAGAG
		R: AGCTGACCCCAGCAATACTT
*CCND2*	102180657	F: GGGCAAGTTGAAATGGAA
		R: TCATCGACGGCGGGTAC
*Bax*	100846984	F: GGGCAAGTTGAAATGGAA
		R: TCATCGACGGCGGGTAC
*Bcl-2*	100861254	F:ATGTGTGTGGAGAGCGTCAA
		R: CCTTCAGAGACAGCCAGGAG

Note: F represents forward primer; R represents reverse primer.

**Table 2:** RNA oligonucleotide sequence information.

Genes	Sequence Name	Sequence (5′–3′)
miR-101	Negative control	UUCUCCGAACGUGUCACGUTT (sense)
		ACGUGACACGUUCGGAGAATT (antisense)
	Mimics	CAGUACUGUGAUAACUGAATT (sense)
		UUCAGUUAUCACAGUACUGUA (antisense)
	Inhibitor-negative control	CAGUACUUUUGUGUAGUACAA
	Inhibitors	UUCAGUUAUCACAGUACUGUA

## Supplementary Materials

The following supporting information can be downloaded at: https://www.mdpi.com/article/10.3390/genes13061035/s1, Figure S1: Sequence alignment analysis of the coding sequence of wild DUSP1 in the vector-matched coding sequence of *DUSP1* in the *DUSP1* database; Figure S2: Sequence alignment analysis of the coding sequence of Mut-*DUSP1* in the vector-matched coding sequence of *DUSP1* in the *DUSP1* database.
