# Supplementary material for: Effect of miR-101 on the Proliferation and Apoptosis of Goat Hair Follicle Stem Cells
Source: Genes (Basel). 2022 Jun 9;13(6):1035. doi: 10.3390/genes13061035 (PMC9222262; doi:10.3390/genes13061035)
Supplement: Supplementary file 1 [file genes-13-01035-s001.zip › genes-1686959-supplementary.pdf]

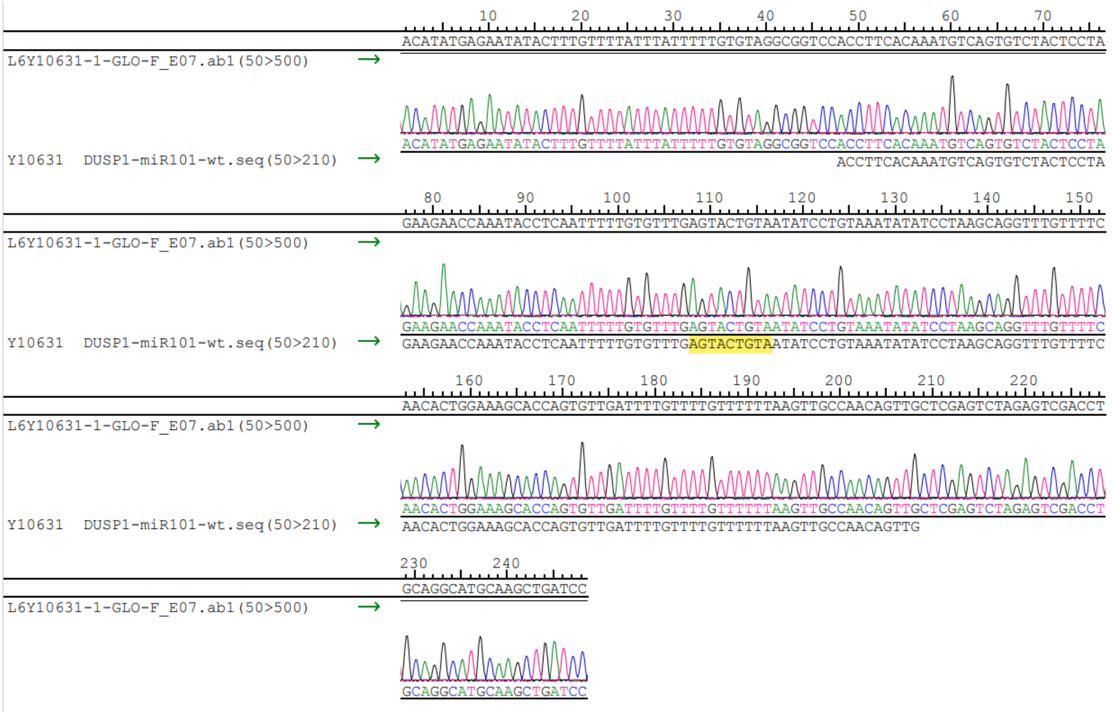

Figure S1: Sequence alignment analysis of the coding sequence of wild-*DUSP1* in vector matched the coding sequence of *DUSP1* in the *DUSP1* database. The coding sequence in wild-*DUSP1* vector targets miR-101 with bright light is consistent with the coding sequence of *DUSP1* in the *DUSP1* database, which means the wild-*DUSP1* plasmid vector is available.

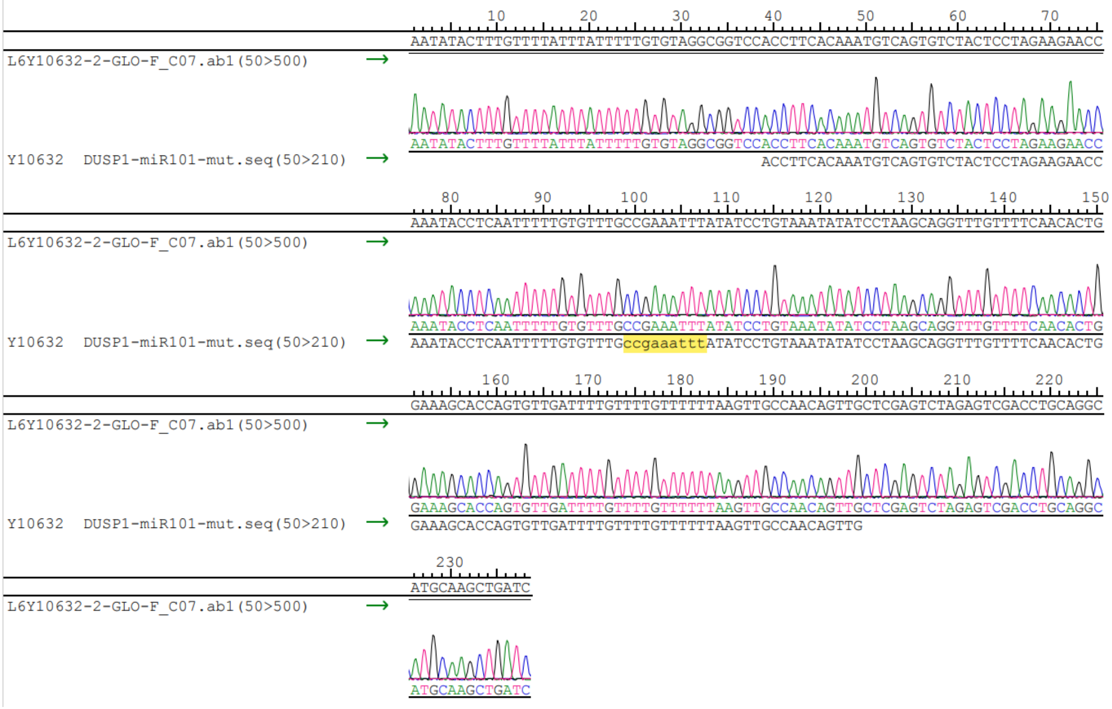

Figure S2: Sequence alignment analysis of the coding sequence of Mut-*DUSP1* in vector matched the coding sequence of DUSP1 in the DUSP1 database. The coding sequence in mut-*DUSP1* vector targets miR-101 with bright light is consistent with the displaced sequence of *DUSP1*, which means the wild-*DUSP1* plasmid vector is successfully constructed.
